# Supplementary material for: Development of a conceptual model for evaluating new non-curative and curative therapies for sickle cell disease
Source: PLoS One. 2022 Apr 28;17(4):e0267448. doi: 10.1371/journal.pone.0267448 (PMC9049306; doi:10.1371/journal.pone.0267448)
Supplement: S1 Appendix — (DOCX) [file pone.0267448.s001.docx]

**APPENDIX**

Development of a Conceptual Model for Evaluating New Non-Curative and Curative Therapies for Sickle Cell Disease

**Table A1:** List of disease attributes included in the conceptual model

| Disease Dimension | Attributes |
| --- | --- |
| Pain | Chronic pain, Vaso-occlusive pain |
| Acute Events | Stroke, Acute chest syndrome, Infections, Priapism, Splenic disease, Bacteremia and sepsis, Myocardial infarction, Acute renal failure, Acute anemia, Multi-organ failure, Fever, Dactylitis |
| Chronic Disorders | Fatigue, Chronic renal disease, Liver disease and hepatobiliary complications, Asthma, Avascular necrosis & bone damage, Chronic lung diseases, Depression and unspecified psychosis, Ocular complications, Sleep disordered breathing and nocturnal hypoxemia, Pulmonary hypertension and cardiovascular diseases, Leg ulcers, Cognitive impairment |
| Treatment Complications | *Hydroxyurea*: Leukopenia, Thrombocytopenia, and Oligospermia/Azospermia  *Transfusion*: Iron overload, Transfusion reactions, Infection  *HSCT*: Graft versus host disease, Graft failure, Bronchiolitis obliterans, Osteoporosis, Iron overload, Depression, Post-transplant lymphoproliferative, Posterior reversible encephalopathy syndrome, Secondary malignancy  *Gene Therapy*: Graft failure, Bronchiolitis obliterans, Osteoporosis, Iron Overload, Depression, Post-transplant lymphoproliferative, Posterior reversible encephalopathy syndrome, Secondary malignancy |

**Table A2:** Impact Inventory

| **Sector** | **Type of Impact** | **Included in This Analysis from…Perspective?** | | **Sources of Evidence** |
| --- | --- | --- | --- | --- |
|  |  | Health Care Sector | Societal |  |
| **Formal Health Care Sector** | | | | |
| **Health** | *Health outcomes (effects)* | | | |
|  | Longevity effects | Y | Y | Health claims |
|  | Health-related quality-of-life effects | Y | Y | SCCRIP |
|  | Other health effects (eg, adverse events and secondary transmissions of infections) | Y | Y | Health claims |
|  | *Medical costs* | | | |
|  | Paid for by third-party payers | Y | Y | Health claims |
|  | Paid for by patients out-of-pocket | Y | Y | Health claims |
|  | Future related medical costs (payers and patients) | Y | Y | Health claims |
|  | Future unrelated medical costs (payers and patients) | Y | Y | Reference estimates^42^ |
| **Informal Health Care Sector** | | | | |
| **Health** | Patient-time costs | NA | Y | SCCRIP and ATUS^41^ |
|  | Unpaid caregiver-time costs | NA | Y | Reference estimates^6^ |
|  | Transportation costs | NA | Y | SCCRIP and ATUS^41^ |
| **Non−Health Care Sectors** | | | | |
| **Productivity** | Labor market earnings lost | NA | Y | SCCRIP and ATUS^41^ |
|  | Cost of unpaid lost productivity due to illness | NA | Y | SCCRIP and ATUS^41^ |
|  | Cost of uncompensated household production | NA | Y | SCCRIP and ATUS^41^ |
| **Consumption** | Future consumption unrelated to health | NA | N |  |
| **Social Services** | Cost of social services as part of intervention | NA | N |  |
| **Legal or Criminal Justice** | Number of crimes related to intervention | NA | N |  |
|  | Cost of crimes related to intervention | NA | N |  |
| **Education** | Impact of intervention on educational achievement of population | NA | N |  |
| **Housing** | Cost of intervention on home improvements (eg, removing lead paint) | NA | N |  |
| **Environment** | Production of toxic waste pollution by intervention | NA | N |  |

SCCRIP: Sickle Cell Clinical Research and Intervention Program; ATUS: American Time Use Survey

Health claims data are from the Truven MarketScan commercial claims database, CMS Medicaid Analytic eXtract database and Medicare Fee-For-Service database

**Table A3:** Diagnostic and procedure codes used to define acute events, chronic disorders, treatment complications, treatments and procedures in health claims databases

| **Condition** | **ICD9** | **ICD10** |
| --- | --- | --- |
| **ACUTE EVENTS** | | |
| **Vaso-occlusive pain episodes** | 282.62, 282.64, 282.69, 282.42, 282.64, 282.62, 282.69 | D57.0, D57.00, D57.21, D57.219, D57.41, D57.419, D57.81, D57.819, D57.00, D57.219, D57.419, D57.819 |
| **Stroke** | 433, 434, 438, 437.5, 431, 433.11, 433.21, 433.81, 433.91, 434.1, 434.11, 434.91, 435.8, 435.9 | I63.50, I63.9, I63.89, I67.83, I67.89, I67.9, I69.80, G46.8, I67.5, G45, G45.8, G45.9, G46, G46.1, G46.2, G46.5, G46.6, G46.7, G46.8, I61, I61.1, I61.2, I61.3, I61.4, I61.5, I61.6, I61.8, I61.9, I63.00, I63.02, I63.011, I63.012, I63.119, I63.12, I63.131, I63.132, I63.139, I63.19, I63.20, I63.211, I63.212, I63.213, I63.219, I63.22, I63.231, I63.232, I63.233, I63.239, I63.29, I63.30, I63.311, I63.312, I63.313, I63.319, I63.321, I63.322, I63.323, I63.329, I63.331, I63.332, I63.333, I63.339, I63.341, I63.342, I63.343, I63.349, I63.39, I63.40, I63.411, I63.412, I63.413, I63.419, I63.421, I63.422, I63.423, I63.429, I63.431, I63.432, I63.433, I63.439, I63.441, I63.442, I63.443, I63.449, I63.49, I63.50, I63.511, I63.512, I63.513, I63.519, I63.521, I63.522, I63.523, I63.529, I63.531, I63.532, I63.533, I63.539, I63.541, I63.542, I63.549, I63.59, I63.6, I63.8, I63.9, I66.01, I66.02, I66.03, I66.09, I66.11, I66.12, I66.13, I66.19, I66.21, I66.22, I66.23, I66.29, I66.3, I66.8, I66.9, I67.841, I67.848, I67.89 |
| **Fever** | 780.60, 780.61 | R50.81, R50.9 |
| **Splenic Disease** | 298.52, 789.2 | D57.02, D57.212, D57.412, D57.812, R16.1, D73.5 |
| **Priapism** | 607.3 | N48.30, N48.32, N48.39 |
| **Dactylitis** | 686 | L08.9 |
| **Acute chest syndrome** | 517.3, 480, 481, 482.0, 483, 484, 485, 486.0 | D57.01, D57.211, D9D57.411, D57.811, J12.0, J12.1, J12.2, J12.81, J12.89, J12.9, J13, J15.0, J15.7, J16.0, J16.8, B25.0, A37, A22.1, B44.0, J17, J18.0, J18.9 |
| **Myocardial infarction** | 410, 410.9, 411.81, 410.01, 410.11, 410.21, 410.31, 410.41, 410.51, 410.61, 410.71, 410.81, 410.91 | I21.9, I21.A1, I21.A9, I21.01, I21.02, I21.09, I21.11, I21.19, I21.21, I21.29, I21.3, I21.4, I22, I22.1, I22.2, I22.8, I22.9 |
| **Infections** | 036, 322.9, 415, 079.99, 465.9, 481, 482.2, 483.0, 486, 599.0, 599.9, 460.0-466.0, 730, 730.0, 730.00, 730.01, 730.02, 730.03, 730.04, 730.05, 730.06, 730.07, 730.08, 730.09, 730.2, 730.20, 730.21, 730.22, 730.23, 730.24, 730.25, 730.26, 730.27, 730.28, 730.29, 730.8, 730.80, 730.81, 730.82, 730.83, 730.84, 730.85, 730.86, 730.87, 730.88, 730.89, 590.10, 590.11 | A04.8, A49.2, A49.8, A49.9, B95.3, B96.0, G00.8, G00.9, G01, G03.8, G03.9, J06.9, J13, J14, J15.7, J20.0, J20.1, J22, L08.89, L08.9, M46.20-28, M86.00, M86.08, M86.09, M86.10, M86.28, M86.29, M86.8X0, M86.8X8, M86.8X9, M86.9, N39.0, M86.00, M86.011, M86.012, M86.019, M86.021, M86.022, M86.029, M86.031, M86.032, M86.039, M86.041, M86.042, M86.049, M86.051, M86.052, M86.059, M86.061, M86.062, M86.069, M86.071, M86.072, M86.079, M86.08, M86.09, M86.10, M86.111, M86.112, M86.119, M86.121, M86.122, M86.129, M86.131, M86.132, M86.139, M86.141, M86.142, M86.149, M86.151, M86.152, M86.159, M86.161, M86.162, M86.169, M86.171, M86.179, M86.18, M86.19, M86.9, N10 |
| **Bacteremia and Sepsis** | 038.0, 771.81, 771.83, 785.52, 790.7, 795.3, 995.90, 995.91, 995.92, 995.93, 995.94 | A02.1, A40.0, A40.1, A40.3, A40.8, A40.9, A41.01, A41.02, A41.1, A41.2, A41.3, A41.4, A41.50, A41.51, A41.52, A41.59, A41.81, A41.89, A41.9, P36.0, P36.10, P36.19, P36.2, P36.30, P36.39, P36.4, P36.5, P36.8, P36.9, R65.20, R65.21, 78.81 |
| **Acute renal failure** | 584.9, 599.71 | N17.0, N17.1, N17.2, N17.8, N17.9, R31.0 |
| **Multi-organ failure** | 995.92 | R65.20 |
| **Acute anemia** | 079.83, 284.12, 285.3 | B34.3, D57.212, D57.412, D57.812, D57.819, D60.1, D61.810, D61.811, D64.81 |
| **CHRONIC DISORDERS** | | |
| **Chronic pain** | 338.4, 338.29, 729.2 | G89.29, G89.4, M79.2 |
| **Leg Ulcers** | 707.19 | I70.231-75 |
| **Fatigue** | 780.7, 780.71, 780.79 | R53.81, R53.82, R53.83 |
| **Asthma** | 493, 493.0, 493.90, 493.91, 493.92, 493.9, 786.07, 493.00, 493.01, 493.02, 493.10, 493.11, 493.12, 493.20, 493.21, 493.22, 493.81, 493.82, 493.90, 493.91, 493.92 | J45.20, J45.21, J45.22, J45.30, J45.31, J45.32, J45.40, J45.41, J45.42, J45.50, J45.51, J45.52, J45.901, J45.902, J45.909, J45.991, J45.998, R06.2, J45.20, J45.21, J45.22, J45.30, J45.31, J45.32, J45.40, J45.41, J45.42, J45.50, J45.51, J45.52, J45.901, J45.902, J45.909, J45.990, J45.991, J45.998 |
| **Hepatobiliary Complications and Liver Disease** | 560.31, 570, 571.6, 571.9, 572.2, 572.3, 572.4, 574.00, 574.01, 574.10, 574.11, 574.20, 574.21, 574.30, 574.31, 574.40, 574.41, 574.50, 574.51, 574.60, 574.61, 574.70, 574.71, 574.80, 574.81, 574.90, 574.91, 575.0, 575.1, 575.3, 575.10, 575.11, 575.12, 575.2, 575.4, 575.6, 576.1, 576.2, 576.3, 576.4, 576.5, 576.8, 571.9, 573.9, 571.9, 573.9 | K56.3, K72.00, K81.0, K81.1, K81.2, K81.9, K80.00, K80.01, K80.10, K80.11, K80.12, K80.13, K80.18, K80.19, K80.20, K80.21, K80.30, K80.31, K80.32, K80.33, K80.34, K80.35, K80.36, K80.37, K80.40, K80.41, K80.42, K80.43, K80.44, K80.45, K80.46, K80.47, K80.50, K80.51, K80.60, K80.61, K80.62, K80.63, K80.64, K80.65, K80.66, K80.67, K80.70, K80.71, K80.80, K80.81, K82.0, K82.1, K82.2, K82.8, K82.9, K82.A1, K82.A2, R93.2, K72.00, K72.01, K74.0, K74.1, K74.2, K75.89, K75.9, K76.89, K76.9, K77, K75.89, K75.9, K76.89, K76.9, K77 |
| **Sleep disordered breathing and nocturnal hypoxemia** | 327.26, 327.23, 799.02, 320.20 | G47.30, G47.10, G47.11, G47.12, G47.13, G47.14, G47.19, R09.02, G47.30 |
| **Cognitive impairment** | 315.09, 315.1, 315.8, 315.9, 317, 318.0, 318.1, 318.2, 331.83, 331.9, 315.8, 3190, 438.0, 783.42, 799.52, 799.53, 799.54, 799.55, 799.59, V40.0, 780.93, 294.9, 799.5 | G31.84, F69, F70, F71, F72, F73, F78, F79, F80.89, F80.9, F81.2, F81.81, F81.9, F88, R41, R41.83, R41.840, R41.841, R41.842, R41.843, R41.844, R41.89, R41.9, R45.87, R62.0, I69.911, I69.912, I69.913, I69.914, I69.11, I69.01, I69.91, I69.21, I69.31, I69.81, G31.84 |
| **Chronic renal disease** | 586, 585.1, 585.2, 585.3, 585.4, 585.5, 585.9, 599.72, 599.70, 403.11, 403.91, 404.12, 404.13, 404.92, 404.93, 580.0, 580.4, 580.81, 580.89, 580.9, 581.0, 581.1, 581.2, 581.3, 581.81, 581.89, 581.9, 582.0, 582.1, 582.2, 582.4, 582.81, 582.89, 582.9, 583.0, 583.1, 583.2, 583.4, 583.6, 583.7, 583.81, 583.89, 583.9, 584.5, 584.6, 584.7, 584.8, 584.9, 585.1, 858.2, 585.3, 585.4, 585.5, 585.6, 585.9, 586, 587, 588.0, 588.1, 588.81, 588.9 | N18.1, N18.2, N18.3, N18.4, N18.5, N18.9, R31.1, R31.21, R31.29, R31.9, I12.0, I12.9, I13.0, I13.10, I13.11, I13.2, M35.04, N00.0, N00.1, N00.2, N00.3, N00.4, N00.5, N00.6, N00.7, N00.8, N00.9, N01.0, N01.2, N01.3, N01.4, N01.5, N01.6, N01.7, N01.8, N01.9, N02.0, N02.1, N02.2, N02.3, N02.4, N02.5, N02.6, N02.7, N02.8, N02.9, N03.0, N03.1, N03.2, N03.3, N03.4, N03.5, N03.6, N03.7, N03.8, N03.9, N04.0, N04.1, N04.2, N04.3, N04.4, N04.5, N04.6, N04.7, N04.8, N04.9, N05.0, N05.1, N05.2, N05.3, N05.4, N05.5, N05.6, N05.7, N05.8, N05.9, N06.0, N06.1, N06.2, N06.3, N06.4, N06.5, N06.6, N06.7, N06.8, N06.9, N08, N15.8, N15.9, N16, N17.0, N17.1, N17.2, N17.8, N17.9, N18.1, N18.2, N18.3, N18.4, N18.5, N18.6, N18.9, N19, N25.0, N25.1, N25.89, N25.9, N26.1, N26.9 |
| **Avascular necrosis & bone damage** | 733.40, 733.41, 733.42, 733.43, 733.49 | M87.00, M87.30, M87.80, M87.88, M87.89, M87.9 |
| **Chronic lung diseases** | 491.21, 493.0, 493.22, 496, 518.89 | J44.9, J44.0, J44.1, J84.17, J84.89, J84.9, J98 |
| **Depression and unspecified psychosis** | 296.20, 296.30, 296.31, 296.32, 296.33, 296.34, 296.35, 296.36, E950-E959, V62.84, 300.00 | F29, F32.9, F33.1, F33.2, F33.3, F33.40, F33.41, F33.42, F33.8, F33.9, F32.89, F33.8, F34.81, F34.89, F34.9, F39, R45.851, T14.91XA, T14.91XD, T14.91XS, F41.9 |
| **Ocular Complications** | 362.29, 364.41, 361.00, 361.01, 361.02, 361.03, 361.04, 361.05, 361.07 | H35.20, H35.21, H35.22, H35.23, H21.00, H21.01, H21.02, H21.03, H33.001, H33.002, H33.003, H33.009, H33.011, H33.012, H33.013, H33.019, H33.021, H33.022, H33.023, H33.029, H33.031, H33.032, H33.033, H33.039, H33.041, H33.042, H33.043, H33.049, H33.051, H33.052, H33.053, H33.059 |
| **Pulmonary hypertension and cardiovascular diseases** | 428.21, 428.22, 428.23, 428.30, 414.9, 798, 786.05, 425.8, 428.1, 428.30, 428.31, 428.32, 428.33, 428.0, 428.1, 428.20, 428.21, 428.22, 428.30, 428.31, 428.32, 428.33, 428.40, 428.42, 428.43, 428.9, 410.00, 410.01, 410.02, 410.10, 410.11, 410.12, 410.20, 410.21, 410.22, 410.30, 410.31, 410.32, 410.40, 410.41, 410.42, 410.50, 410.51, 410.52, 410.60, 410.61, 410.62, 410.70, 410.71, 410.72, 410.80, 410.81, 410.82, 410.90, 410.91, 410.92, 411.0, 411.1, 411.81, 411.89, 412, 413.0, 413.1, 413.9, 414.00, 414.01, 414.2, 414.3, 414.4, 414.8, 414.9, 416.0, 416.8, 416.9 | I50.21, I50.22, I50.23, I50.30, I25.9, I46.1, R06.09, I42.9, I50.1, I50.31, I50.32, I50.33, I50.30, I13.2, I50.1, I50.20, I50.21, I50.22, I50.23, I50.30, I50.31, I50.32, I50.33, I50.40, I50.41, I50.42, I50.43, I50.9, I20.0, I20.1, I20.8, I20.9, I21.01, I21.02, I21.09, I21.11, I21.19, I21.21, I21.29, I21.3, I21.4, I22.0, I22.1, I22.2, I22.8, I2.9, I23.6, I23.7, I23.8, I24.0, I24.1, I24.8, I24.9, I25.10, I25.110, I25.111, I25.118, I25.119, I25.2, I25.5, I25.6, I25.82, I25.83, I25.84, I25.89, I25.9, I27.0, I27.20, I27.21, I27.22, I27.23, I27.24, I27.29, I27.81, I27.9 |

**Figure A1:** Cohort Selection in CMS data

^*^ Patients are censored from the year preceding hematopoietic stem cell transplant (HSCT)

**Figure A2:** Relation between variables included in the predictive indices.


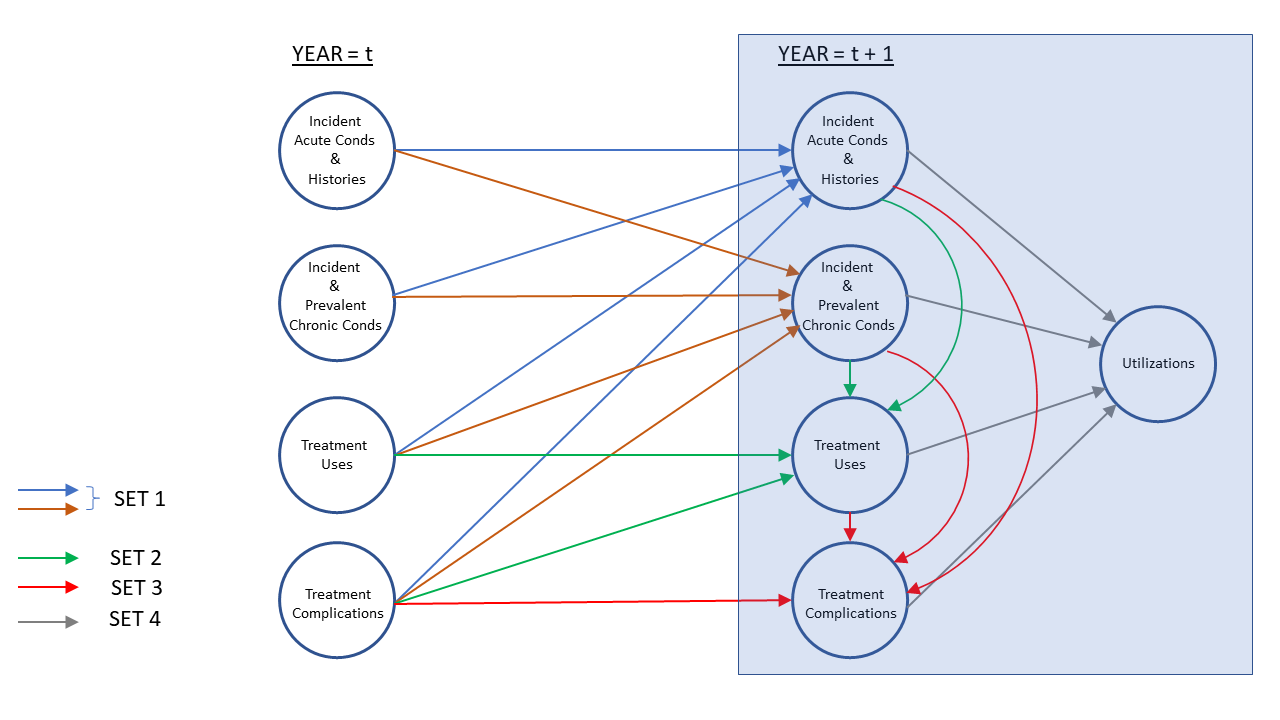


**Set 1:** Predict occurrence of each acute condition, and incidence of each chronic condition

E(Y|X) = G(X = chronic conditions indicators, acute event indicators, & disease history in PREVIOUS year, treatment use in PREVIOUS year, treatment complications in PREVIOUS year, demographics, and interactions with age and sex)

**Set 2:** Predict treatment use – Any hydroxyurea use, Any transfusion use, Any use of transfusions in ≥ 6 distinct months within a 12-month period

E(Y|X) = G(X = chronic conditions indicators, acute event indicators, & disease history in SAME year, treatment use in PREVIOUS year, treatment complications in PREVIOUS year, demographics, and interactions with age and sex)

**Set 3:** Predict occurrence of each treatment complication

E(Y|x) = G(X = chronic conditions indicators, acute event indicators, & disease history in SAME year, treatment use in SAME year, treatment complications reported in PREVIOUS year, demographics, and interactions with age and sex)

**Set 4:** Predict utilizations of inpatient hospitalizations, ED visits, office visits, outpatient visits, long-term care days, number of unique NDC besides NSAID/Opioids, any NSAID/Opioids

E(Y|X) = G(X = chronic conditions indicators, acute event indicators, & disease history in SAME year, treatment use in SAME year, treatment complications in SAME year, demographics, and interactions with age and sex)
