# Supplementary material for: Development of a conceptual model for evaluating new non-curative and curative therapies for sickle cell disease
Source: PLoS One. 2022 Apr 28;17(4):e0267448. doi: 10.1371/journal.pone.0267448 (PMC9049306; doi:10.1371/journal.pone.0267448)
Supplement: Attachment — Submitted filename: Response_to_Reviewers_PlosOne.docx [file pone.0267448.s002.docx]

Reviewer #1: In this article, Anirban Basu and colleagues developed a conceptual model for the evaluation of curative therapies in Sickle Cell Disease (SC). The model was built and discussed using consultations with the patients and health care providers and experts and also by conducting intensive literature review. The model included four chronic events associated with SCD: chronic pain, acute events, chronic conditions and treatment complications. In general, having this model will be helpful in future analysis of SCD-targeting curative therapies. The model’s description is vague and hard to grasp and needs better description. Also, it seems to miss recent therapies that are being incorporated into SCD treatment.

We have tried to provide more details on the model, moving the content in a separate section. We have now clarified that we intentionally did not include any of the recent therapies because out model development protocol is for a comparison/control group based on common care in real world. It is against this control, curative therapies and non-curative newer therapies can be evaluated. In addition, these therapies were not available during the latest year for which CMS data (2016) are released. We have now clarified this throughout the paper.

Major critiques:
1. Please dedicate a section specifically for the description of the model.

Done

2. A visual presentation of the literature review would be helpful.

This has been detailed in a separate publication. We have provided the reference.

3. While blood transfusion and hydroxyurea were considered, no others emerging therapies were included such as voxelotor or crizanlizumab treatments.

Please see our response above as to why emerging therapies were not included as part of our control group.

Minor changes
1. References are not in the PLoS One format.

We have corrected this.

Reviewer #2: The cited work appears well researched, rationale and reproducible. Outcome measures were generally justifiable for addressing the questions posed by the authors.
Patient data retrieval from online databases is an attractive option in view of the project’s mandate. Likewise, the multidisciplinary team proposed should prove beneficial in navigating the nuances of sickle cell disease prognoses and economic/societal impact and feasibility.

A few areas of note arose; these were generally recognized by the authors and highlighted under ‘limitations’ of the study.

Firstly, although the prediction analyses appear robust, the depth of the data (age, gender and region) intended for the prediction of healthcare utilization, health state transitions, adverse events and the likelihood of effective treatment appear somewhat tenuous for the useful simulation of health state fluxes in SCD. The etiology of the disease with its diversity of physiological manifestations should pose unique challenges for the reliable imputation of clinical trajectories. Furthermore, the immunological depression further veils the true impact of SCD-related events, giving rise to above-normal incidences of SCD-independent comorbidities.

We thank the reviewer for raising this point. We discuss some of the limitation of estimating some of these parameters with the data we have. We have some dropped the use of region variables in our analysis due to the need to have sufficient sample sizes for estimating the other parameters. Our plan to employ machine learning techniques can overcome some of these limitations through identifying parsimonious models that are still highly predictive of outcomes. These predictions would be calibrated through split sample techniques. We have added discussions of the limitations in the Discussion section.

Secondly, the appropriateness of roundtable meetings as supplants to consensus from experienced personnel is ambiguous. Again, consequent to the convoluted nature and frequency of the acute and chronic clinical symptomology of SCD, a more prudent ‘real-world’ approach might strengthen the efficacy of a conceptual predictive model.

We agree and that is why we have utilized both input from experience personnel, feedback from patients, and analysis of real-world data to finalize our state space for outcomes, and the universe of features for a predictive model.

Thirdly, the value of the ‘societal’ definition remarked on by the authors is uncertain. The conceptual framework outlined, whilst likely agreeable with the cost-effectiveness ascertainment facet of the work, appears less geared to optimizing the quality of care and clinical outcomes of the target population. The societal view may be skewed away from the advancement of clinical care and patient outcomes towards the tangential sociodemographic and institutional burdens imposed by the patients’ `disease.

Since the goal of this model is primarily to assess cost-effectiveness of emerging therapies, we have taken the societal perspective in line with the guidance laid out by the Second Panel on cost-effectiveness. Generating evidence on the value of use of certain therapies, for certain sub-group of patients, at certain time during their disease trajectory, we hope to inform advancement of clinical care and insurance coverage of appropriate treatments.

Together with the narrowness of the accessible patient data, omission of traditional decision hierarchy, the overall societal benefit of this explicit conceptualization model is not apparent. I support the work and recognize its potential benefit with some minor revisions.

We do use data on the universe of publicly funded individuals with SCD in the US. This helps us to establish models that document the health of this population accurately and conduct population-level and certain sub-population level evaluations. The explicit conceptualization of these models helps make the black-box nature of simulation models more transparent to policy makers. However, our model may not be well suited to inform individual-level clinical care, unless it is calibrated in the future against individual-level clinical trial data. We have added discussions to this end as limitations in the Limitations Section.
